# Assessment of the Properties of AISI 410 Martensitic Stainless Steel by an Eddy Current Method

**DOI:** 10.3390/ma12081290

**Published:** 2019-04-19

**Authors:** Huayu Zhang, Zhiheng Wei, Fengqin Xie, Baohai Sun

**Affiliations:** 1 College of Mechanical and electronic Engineering, Shandong University of Science and Technology, Qingdao 266590, China; weizhiheng2016@163.com; 2 College of Transportation, Shandong University of Science and Technology, Qingdao 266590, China; 3 Yihe Electric Group Co., Ltd., 717 Huaihe West Road, Huangdao District, Qingdao 266590, China; skdmcu@163.com

**Keywords:** AISI 410 martensitic stainless steel, quenching, hardness, eddy current nondestructive testing, microstructure

## Abstract

Based on electromagnetic theory, metallurgical characteristics can be detected by eddy current nondestructive testing technology. In this study, the relationship between the surface microstructure and the eddy current output of martensitic stainless steel AISI 410 was studied using this technology at different quenching temperatures. The mechanical properties include material hardness, microstructure types and microstructural changes after thermal treatment was evaluated. Using Vickers hardness as the surface hardness index of AISI 410 steel, the relationship between eddy current output signal, in terms of impedance and inductance, and sample surface hardness was studied and the effects of different quenching temperatures on the steel’s surface hardness was examined. In addition, the change of microstructure types of AISI 410 steel after thermal treatment was detected by the eddy current nondestructive testing method, and the results were verified by metallographic microscopy.

## 1. Introduction

Martensitic stainless steel (MSS), with its good combination of high strength and ductility, has become a new type of stamping steel, which is widely used in the automotive and general industries and the national defense industry [1,2]. As cast MSS mainly consists of massive ferrite and granular carbide distributed in a ferrite matrix, its wear and fatigue resistance cannot meet certain requirements. To improve the service life of MSS, heat treatment is often applied to improve the surface hardness and other mechanical properties. After quenching, the structure of MSS stainless is composed of martensite, ferrite, and a small amount of carbide. It is the martensite in the steel that improves the steel’s wear resistance and surface strength.

As the permeabilities of ferrite and martensite are different, the compositions and volume fractions of ferrite and martensite are different as a result of different tempering temperatures, which leads to different steel permeabilities or conductivities, such that the nondestructive evaluation of steel microstructure can be realized by detecting changes in permeability, conductivity, and other parameters. An eddy current nondestructive testing method measures changes in the impedance signal of a testing coil to characterize the changes in a material’s metallographic composition, such that it reflects the changes in material hardness. Compared with traditional destructive hardness testing methods, eddy current nondestructive testing can evaluate the hardness of heat-treated materials without destructive sampling and also has the advantages of short analysis time and high efficiency.

Hughes [3] has put forward the concept of eddy current nondestructive testing for the first time, which was the first step for the development of eddy current testing (ECT) technology. Konoplyuk [4] has detected nodular cast iron with different pearlite volume fractions using an eddy current probe, analyzing the linear relationship between the impedance amplitude of the eddy current coil and chemical composition of nodular cast iron, and the chemical composition of nodular cast iron via a fitting equation. Ghanei et al. [5] have evaluated dual-phase steels using ECT, with the testing results linked to different martensite percentages in samples and the method then used to determine martensite percentages in unknown samples. In addition, the method has been used to predict the mechanical properties of various ferrous metals. Zergoug et al. [6] have studied the relationship between mechanical and impedance change by ECT, indicating that this method can detect and quantitatively analyze the mechanical properties of metal materials, such as aluminum and steel without damage. Khan et al. [7] have analyzed the content of pearlite in common carbon steel samples using ECT. Sorger et al. [8] have measured the electrical conductivity distribution of Ti6Al4V, Cu, Pb, and S355 steel treated by friction stirring and AISI304 stainless steel treated by tungsten gas shielded arc welding by ECT and a four-probe method. At the same time, hardness measurements have been performed and electrical conductivity concluded to be better than hardness for distinguishing microstructural changes in processed material. Mercier et al. [9] have analyzed the decarburization depth of SAE 92V45 steel samples by ECT, measured the microhardness of decarburized samples, and studied the correlation between eddy current response signals and hardness. According to the thickness of hardened layers of AISI 1045 steel bar, Kahrobaee et al. [10] have compared microstructural differences and hardness values of samples using multifrequency and multi-output eddy current sensors and obtained the relationship between the output voltage of sensors and hardness of samples. Hashmi et al. [11] have evaluated the eddy current output characteristics of chromium‒vanadium spring steel under different heat treatments, and successfully distinguished the effects of different heat treatments on sample hardness changes.

The present results showed that the main factor causing differences in the eddy current output signal is microstructural differences, which also determined the macroscopic mechanical properties, such as hardness, and the electromagnetic properties, including conductivity and permeability. Through metallographic microscopy, the same batch of AISI 410 MSS specimens was found to exhibit different martensitic content after different quenching treatments and differences in surface metallographic structure in different samples led to different output responses from the eddy current sensor as well as different surface harnesses. By analyzing the related variations, the relationship between eddy current response signal, as impedance and inductance, and surface hardness of AISI 410 MSS at different quenching temperatures was established.

## 2. Experimental Procedures

### 2.1. Material Composition

In this study, cast AISI 410 MSS was used as the test material, whose structure consisted of massive ferrite and granular carbide distributed in a ferrite matrix. The chemical composition is shown in Table 1.

### 2.2. Heat Treatment

In this test, eight samples were taken from an AISI 410 MSS ingot, sequentially numbered 1 to 8, and the basic size at 50 mm × 40 mm × 4 mm (length × width × thickness, respectively). When the heating temperature was below the critical temperature Ac1 (811.7 °C), Austenite transformation did not occur in the original structure, which is to say the original structure remained unchanged. Samples with different volume fractions of martensite phase were obtained by heating samples at 800, 820, 840, 860, 880, 900, 930, and 970 °C for 1 h and then quenched in mineral oil. According to the heat treatment process, specimens with different microstructures were obtained (Table 2).

### 2.3. Eddy Current Detection System

**Figure 1:**
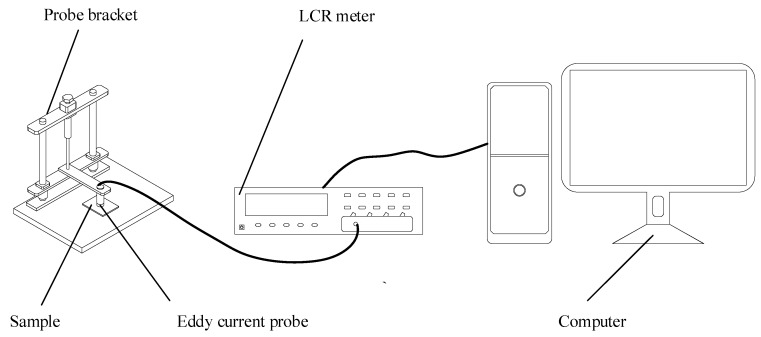
Schematic of eddy current testing setup.

The eddy current detection system was composed of an eddy current detecting probe (Agilent 4294A precision impedance analyzer, Agilent Technologies, Inc., Santa Clara, CA, USA) and a computer (Figure 1), with detection carried out at room temperature (21 °C). An air core cylinder coil was used in the detecting probe, with a height and inner and outer diameters of 8, 4, and 8 mm, respectively. In addition, the insulated copper wire diameter was 0.3 mm. When detection is performed on a specimen surface, good impedance resolution was obtained by setting the coil excitation to 1 kHz sinusoidal Alternating Current and the distance between probe and sample approximately zero.

With the eddy current sensor, a transformer model was used to explain the coupling relationship between the eddy current sensor’s detection coil and the sample. The principle of eddy current detection is that, if a conductor is in a variable magnetic field or is moving relative to the magnetic field, then an interior induced current is generated. When the alternating current passes through the coil, the conductor will experience a changing magnetic field, thus inducing an internal eddy current. The alternating magnetic field produced by the eddy current is opposite to the coil’s magnetic field, thus reducing the magnetic field strength of the coil and affecting the coil’s current, such that the external impedance and inductance of the coil are altered. Some sample characteristics can be obtained by detecting these changes.

According to the literature [12], the main factors causing impedance changes are the conductivity and permeability of the tested material, the coil’s excitation frequency and the lift-off height between the excitation coil and tested material can be expressed as

(1) Z,L=f(x,ρ,μ,f),

where *h* is the lift-off height between the excitation coil and tested material, *μ* the conductivity, *ρ* permeability, and *f* the excitation frequency. When the *h* and *f* are constant, only the permeability and conductivity affect the coil’s impedance and inductance, such that the metallurgical properties of the sample can be distinguished by the changes of impedance and inductance. Equation (1) can be rewritten as

(2) Z,L=f(ρ,μ).

The equivalent inductance *L* and impedance *Z* of the detection coil were recorded by computer and the normalized impedance *Z*/*Z*_0_ obtained by dividing the equivalent *Z* by the empty coil *Z*_0_ of each test block. Similarly, the normalized inductance (*L*/*L*_0_) was obtained by dividing the equivalent *L* by the empty coil *L*_0_.

### 2.4. Microstructure of AISI 410 Steel Samples

**Figure 2:**
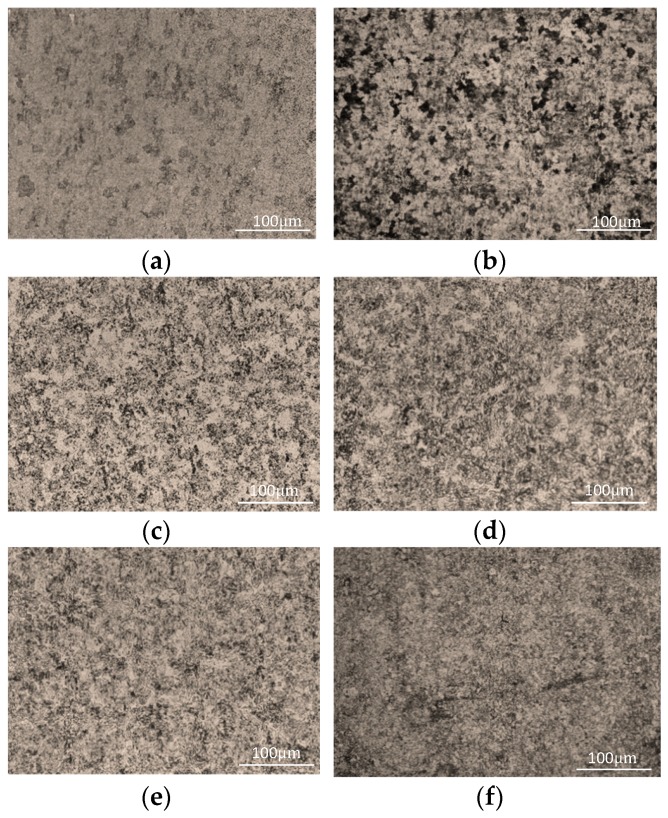

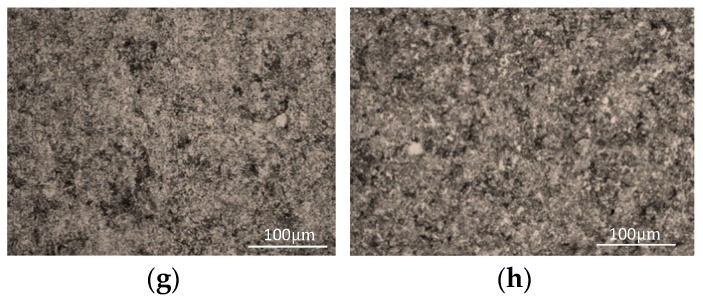
Microstructure of AISI 410 steel treated at different temperatures for 1 h and then oil quenched at (**a**) 800 °C, (**b**) 820 °C, (**c**) 840 °C, (**d**) 860 °C, (**e**) 880 °C, (**f**) 900 °C, (**g**) 930 °C, and (**h**) 970 °C, respectively.

Quenched specimens were cut into metallographic samples, which were polished using silicon papers with 320, 800, 1200, 2500, and 5000 grit. Then the deformed layer produced by initial polishing is removed by magnesium oxide for fine polishing, and etched with aqua regia solution (concentrated hydrochloric and nitric acids, 3/1 v/v). Each sample’s microstructure was finally observed using an Axio Lab, equipped with an A1 Mat metallographic microscope (Carl Zeiss AG, Oberkochen, Germany; Figure 2).

### 2.5. Hardness Test of AISI 410 Steel

**Figure 3:**
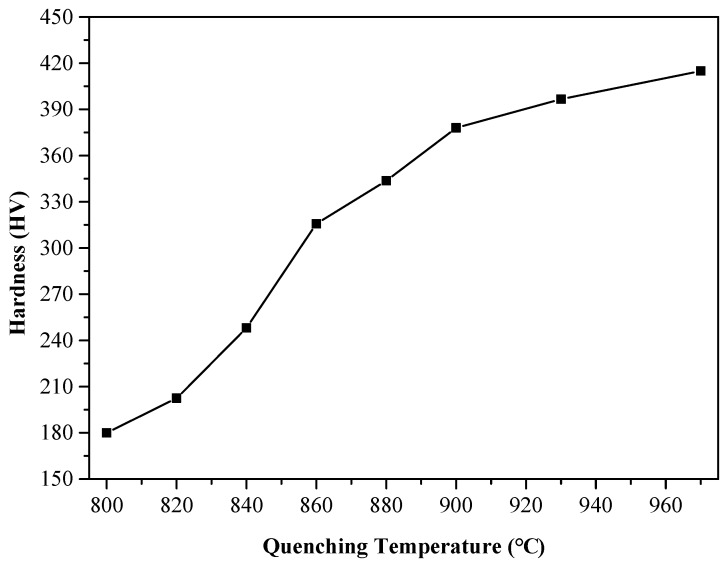
Variation of hardness with increase in quenching temperature.

The hardness of the polished surface of the eight samples was measured by a FM-700 Vickers Hardness Tester (Beijing TIME High Technology Ltd., Beijing, China), controlled by a PC to <100 g loads and loading time of 15 s. During hardness measuring, each sample was measured eight times under the same conditions. After removing the maximum and a minimum values, the arithmetic hardness average was computed (Figure 3). It can be seen that the sample Vickers hardness was increased with increased quenching temperature in this temperature range.

## 3. Results and Discussion

### 3.1. Microstructure Characteristics

Samples’ microstructures differed when obtained at different quenching temperatures (Figure 2). When the heating temperature exceeded the critical temperature Ac1 (811.7 °C), ferrite began to transform into austenite, which rapidly cooled into martensite phase due to the quenching treatment. When the quenching temperature was 800 °C, the microstructure after quenching was fine granular carbides, which were evenly dispersed on the bulk ferrite matrix (Figure 2a), and the microstructure observed to be composed of fine martensite and ferrite (Figure 2b–f). With increased quenching temperature, martensite increased and ferrite decreased, such that it was easy to see that, if the quenching temperature rose to 930 °C, the size of martensite continued increased and ferrite decreased while also distributed in strips (Figure 2g). When the quenching temperature was 970 °C, there was no clear massive ferrite in the structure and the main component was lath martensitic (Figure 2h). If the heating temperature exceeded the critical temperature Ac1 (811.7 °C), austenite was transformed into lath martensite after quenching and the microstructure mainly composed of martensite, ferrite, and a small amount of carbides, with the ferrite content remaining unchanged during quenching. After quenching, the content of martensite increased and ferrite decreased with increased quenching temperature. The Vickers hardness of martensite was much higher than that of ferrite and, thus, increased martensite content led to increased Vickers hardness. Therefore, sample Vickers hardness increased with increased quenching temperature (Figure 3).

### 3.2. Relationship between Electromagnetic Characteristics and Eddy Current Outputs

**Figure 4:**
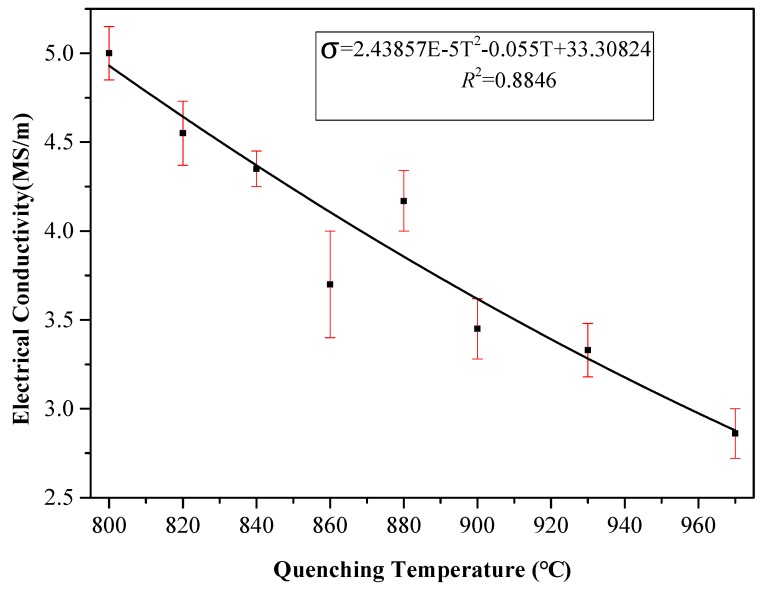
Variation in electrical conductivity with increased quenching temperature.

From analysis of sample microscopic characteristics, it was seen that, in the experimental temperature range, with increased quenching temperature, the greater the martensite content the less the ferrite content. As a result, the grain boundary increased and the average free path of electrons decreased. Therefore, sample conductivity decreased with increased quenching temperature. In addition, quenching treatment could have fixed the vacancy concentration of metal at high temperature, resulting in residual resistance [13]. With increased quenching temperature, the vacancy concentration and residual resistance increased and conductivity decreased [14,15,16]. With increased quenching temperature, the martensite content increased and ferrite content decreased (Figure 2). As the martensite grain size was smaller than that of ferrite, the smaller was the grain size in the sample’s microstructure, and the more internal grain boundaries in a unit area of the sample. This reduced the material’s conductivity and increased its hardness, which is consistent with the change trend of the hardness and conductivity of the sample obtained in our experiment (Figure 3 and Figure 4). The relationship between quenching temperature and conductivity *σ* of the quenched samples was obtained by measuring their conductivity with a ST2263 digital four-probe tester (Suzhou Jingge Electronic Co., Ltd., Suzhou, China), and the correlation coefficient was *R*^2^ = 0.8846. The results showed that sample conductivity decreased with increased quenching temperature, which was consistent with the above analysis results.

The surface microstructure of quenched samples was mainly composed of martensite and ferrite. Martensite transformation occurred in the quenching process, which resulted in high-density dislocations, which not only hindered distortions caused by interstitial atoms but also led to pinning of magnetic domain walls [17,18]. Therefore, domain wall motion was limited, which required a higher reverse field to remove the domain walls and contributed to higher energy loss. As a result, coercivity and hysteresis losses increased while permeability decreased [19,20].

Reaction magnetic fields produced by ferromagnetic materials under low frequency alternating magnetic fields are essentially the same as eddy current reaction magnetic fields formed by high frequency electromagnetic fields in conductive metal materials. That is to say, the magnitude and phase of reaction magnetic fields caused by ferromagnetic materials are closely related to material permeability (material conductivity effects are neglected here as they were very weak). Therefore, the sample eddy current response signals reflect the microcharacteristics. The tested material, AISI 410 stainless steel, was dual-phase steel and the microstructure composed of martensite and ferrite. In the experiment, with increased quenching temperature, martensite content increased, which meant that ferrite content decreased. Therefore, the hardness and eddy current output signal of the material did not only reflect the martensite volume fraction but also that of ferrite in the material’s microstructure. As the permeability of martensite phase was less than that of ferrite phase [10,21], martensite phase permeability decreased as martensite percentage increased. According to reports regarding Equations (3)–(5) in the literature [5], it was concluded that decreased permeability leads to decreased equivalent impedance of the eddy current detection coil.

(3) $$L=\mu N^{2}A/l$$

(4) $$X_{L}=2\pi fL$$

(5) $$Z=\sqrt{X_{L}^{2}+R^{2}},$$

where *L* is the self-inductance coefficient, *μ* the magnetic permeability, *N* the number of turns in the coil, *A* the cross-sectional area, l the length of the coil, *X_L_* the inductive resistance, *f* the excitation frequency, R the resistance, and *Z* the impedance.

**Figure 5:**
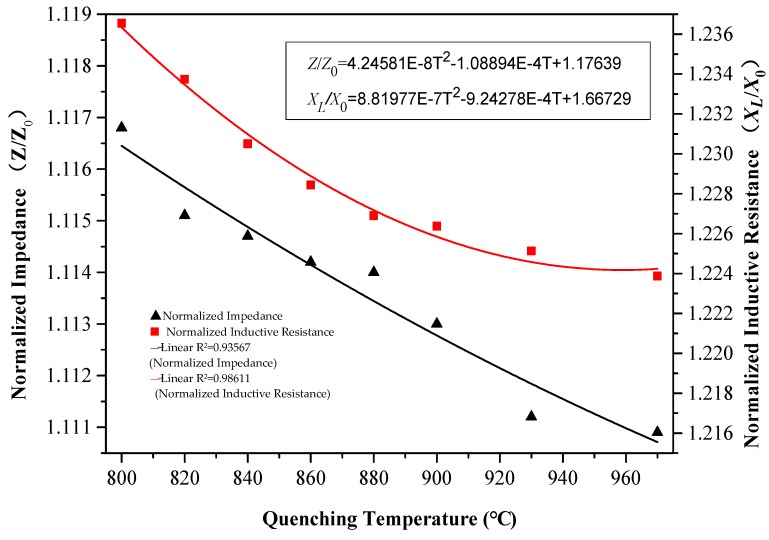
Variation of normalized impedance and normalized inductive resistance with increased quenching temperature.

According to Equations (3)–(5), with decreased permeability, the coil’s self-inductance coefficient decreased and the output of inductance and impedance correspondingly decreased. It should be pointed out that, in ferromagnetic materials, the effect of μ or $X_{L}$ on impedance output is greater than that of resistance *R* [10,22,23]. Thus, the eddy current output, as impedance and inductance, decreased with increased quenching temperature and there was good correlation between eddy current output and changes in quenching temperature (*R*^2^ > 0.93, Figure 5).

### 3.3. Relationship between Hardness and Outputs

**Figure 6:**
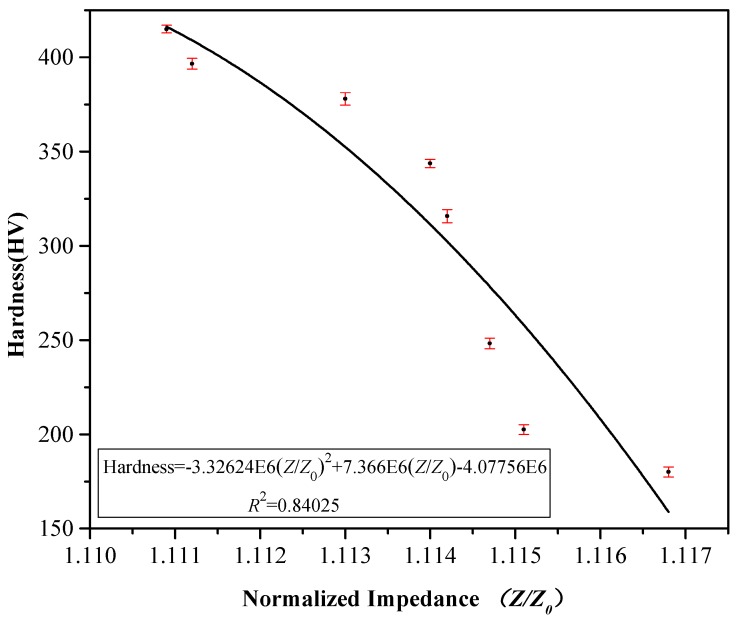
Relation between normalized impedance and hardness.

**Figure 7:**
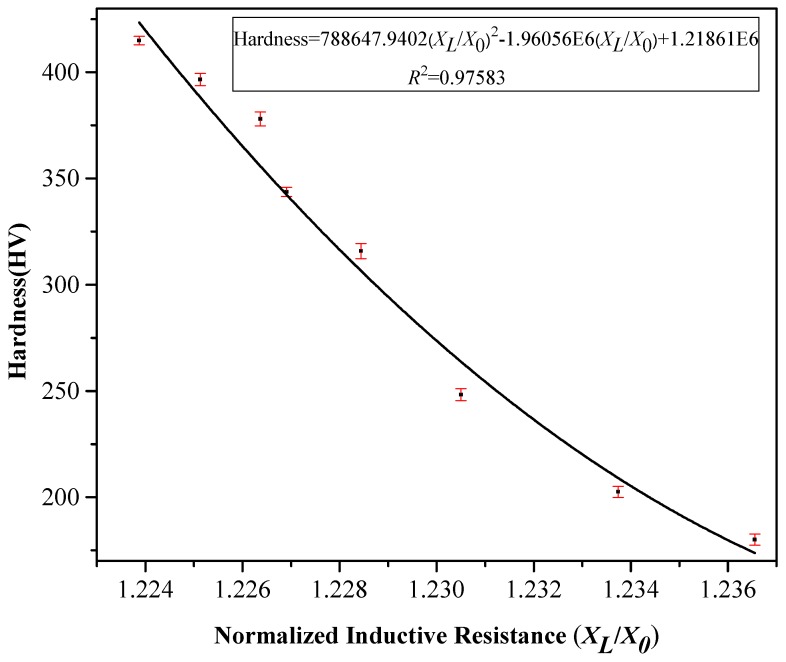
Relationship between the normalized inductive resistance and the hardness.

The hardness of eight samples measured using a Vickers hardness tester was compared with the experimental eddy current output of the same samples measured by the eddy current testing system (Figure 3 and Figure 5, respectively). A correlation was observed between the normalized impedance output and Vickers hardness (Figure 6) and a correlation also observed between the normalized inductance and Vickers hardness value (Figure 7).

Through analysis of sample microstructures, the sample surface hardness was concluded to increase with increased martensite content and that microstructural changes also directly affected the materials, which affected the electromagnetic properties. The eddy current output was observed to decrease with increased sample martensite percentage and thus the relationship between hardness and impedance output was obtained. From the correlation between impedance output and Vickers hardness (*R*^2^ = 0.84025) and the correlation between coil inductance and Vickers hardness (*R*^2^ = 0.97583), it was seen that the latter relationship was stronger than the former (Figure 6 and Figure 7).

The above experiment showed that the detection results from the eddy current sensor were consistent with the microstructural analysis, which verified the predictive reliability of this eddy current testing method. Eddy current testing provided rapid and nondestructive testing of steel hardness and can be expected to be applied in future engineering practices. 

## 4. Conclusions

In this study, the steel sample microstructure was analyzed regarding the effects on the hardness and eddy current output at different quenching temperatures using eddy current nondestructive testing. This eddy current nondestructive testing technology was found to be an effective method for determining the hardness of AISI 410 MSS after thermal treatment. The results showed that an increased martensite volume fraction led to decreased magnetic permeability and eddy current output but increased material hardness. The relationship between steel hardness after quenching and the eddy current output signal was obtained by analyzing the electromagnetic characteristics of different microstructures. In addition, eddy current, metallographic, and hardness testing experiments confirmed that there was good monotonicity and a correlation between eddy current output and changes in steel microstructure and surface hardness. The quadratic fitting correlation coefficient of normalized impedance and hardness is *R*^2^ = 0.84, and the quadratic fitting correlation coefficient of normalized inductive resistance and the hardness is *R*^2^ = 0.98. This technique was helpful for distinguishing and classifying the surface hardness of this steel after quenching at different temperatures.

## Figures and Tables

**Table 1:** Chemical composition of AISI 410 MSS (mass fraction, %).

C	Mn	Si	P	S	Cr	Ni	Fe
0.094	0.42	0.40	0.017	0.004	13.35	0.35	Balance

**Table 2:** Quenching treatment in experiment.

Sample Number	Quenching Temperature	Holding Time	Quenching Method	Main Microstructure
1	800 °C	1 h	Oil quenching	F
2	820 °C	1 h	Oil quenching	M + F
3	840 °C	1 h	Oil quenching	M + F
4	860 °C	1 h	Oil quenching	M + F
5	880 °C	1 h	Oil quenching	M + F
6	900 °C	1 h	Oil quenching	M + F
7	930 °C	1 h	Oil quenching	M + F
8	970 °C	1 h	Oil quenching	M
